# Ablation of symptomatic uterine fibroids with the Mirabilis system for rapid noninvasive ultrasound-guided high-intensity focused ultrasound (HIFU): a prospective observational clinical study

**DOI:** 10.1007/s11547-025-01972-6

**Published:** 2025-03-10

**Authors:** Tolga Tonguc, Oleksandr Savchenko, Olga Ramig, Judith M. Stader, Franziska Kießling, Jim Küppers, Eva K. Egger, Marcus Thudium, Patrick Martin, Wayne Poll, Hans H. Schild, Rupert Conrad, Markus Essler, Alexander Mustea, Holger M. Strunk, Milka Marinova

**Affiliations:** 1 Department of Radiology/Neuroradiology, University Hospital Bonn, University of Bonn, Bonn, NRW Germany; 2 Department of Nuclear Medicine, University Hospital Bonn, University of Bonn, NRW, Venusberg-Campus 1, 53127 Bonn, Germany; 3 Department of Gynaecology and Gynaecological Oncology, University Hospital Bonn, University of Bonn, Bonn, NRW Germany; 4 Department of Anaesthesiology, University Hospital Bonn, University of Bonn, Bonn, NRW Germany; 5 Nalu Medical, Inc, Carlsbad, CA USA; 6 Applied Science Management, LLC, Pismo Beach, CA USA; 7 Department of Psychosomatic Medicine, University Hospital Muenster, University of Muenster, Muenster, NRW Germany; 8 University of Bonn, Bonn, NRW Germany

**Keywords:** High-intensity focused ultrasound, Mobile device, Shell ablation, Mirabilis system, Fibroid-associated symptoms

## Abstract

**Objectives:**

Uterine fibroids often lead to symptoms that negatively impact health-related quality of life (HRQOL). High-intensity focused ultrasound (HIFU) has emerged as a promising noninvasive treatment for reducing fibroid size and symptoms. The Mirabilis system for ultrasound (US)-guided HIFU introduces a novel technique known as ‘shell ablation’. This study evaluates the feasibility and efficacy of Mirabilis in a clinical setting, focusing on clinical outcomes.

**Materials and methods:**

Sixteen patients with 23 uterine fibroids were treated with the Mirabilis system. Follow-up assessments included US and MRI at baseline, 6 weeks, 3, 6 and 9 months, and 1 year after HIFU. Changes in symptoms and QOL were evaluated using the Uterine Fibroid Symptom and HRQOL Questionnaire.

**Results:**

A significant reduction in fibroid volume was observed after HIFU (baseline 182.1 ± 49.3 ml; 1 year: 76.0 ± 37.9 ml, p < 0.001). The symptom severity score significantly declined (baseline 57.2 ± 3.8; 1 year: 30.2 ± 4.9, p < 0.001), correlating with a significant improvement in HRQOL (baseline 47.0 ± 3.9, 1 year: 71.8 ± 5.3, p < 0.001).

**Conclusion:**

HIFU with the portable Mirabilis system is a feasible and safe noninvasive treatment for symptomatic uterine fibroids in an outpatient setting. This approach allows efficient and rapid ablation even for large fibroids, significantly reducing fibroid volume and symptoms.

## Introduction

Uterine fibroids/leiomyomas are benign neoplasms of the uterine smooth muscles and the most common pelvic tumors in women of reproductive age [1]. Fibroid-associated symptoms are present in about 30% of patients between the ages of 30 and 50 years [1–3]. These symptoms often impair the patient’s quality of life (QOL) and encompass pelvic pain during menstruation (dysmenorrhea), excessive menstrual bleeding (hypermenorrhea) or heavy menstrual periods (menorrhagia) possibly leading to chronic anemia [4–6]. Moreover, very large or numerous uterine leiomyomas can cause urinary urgency and constipation due to compression and may have a negative impact on reproductive outcome [5–7]. As the majority of affected patients are women of childbearing age, minimally invasive and noninvasive uterus-preserving treatment options have become increasingly important as alternatives to conventional approaches like laparoscopic or hysteroscopic myomectomy and laparoscopic hysterectomy [5, 8–11]. High-intensity focused ultrasound (HIFU) has emerged as an interesting noninvasive treatment modality. Clinical data demonstrated that HIFU ablation leads to a significant long-term reduction in fibroid volumes, while simultaneously resulting in a decrease in fibroid-related symptoms and an improvement in health-related (HR) QOL [12–18]. Various techniques have been described in the past, and in 2017, the Mirabilis system introduced an innovative technique for ultrasound-guided (US) HIFU ablation [19]. This portable system includes a HIFU applicator with an integrated US device for an US-guided volumetric ablation technique known as shell ablation that allows for shorter treatment durations compared to previous US-guided HIFU systems due to simultaneous ablation of multiple peripheral adjacent focuses.

The system was employed for the first time in clinical routine to treat patients with symptomatic uterine fibroids as part of a prospective observational single-center study conducted at our institution.

The aim of this study was to assess the safety of the treatment by evaluation of ablation-related side effects and assessment of feasibility and efficacy of fibroid shell ablation using the portable HIFU device, focusing on changes in fibroid volume, fibroid-associated symptoms and their impact on HRQOL.

## Material and methods

### Patients

**Fig. 1:**
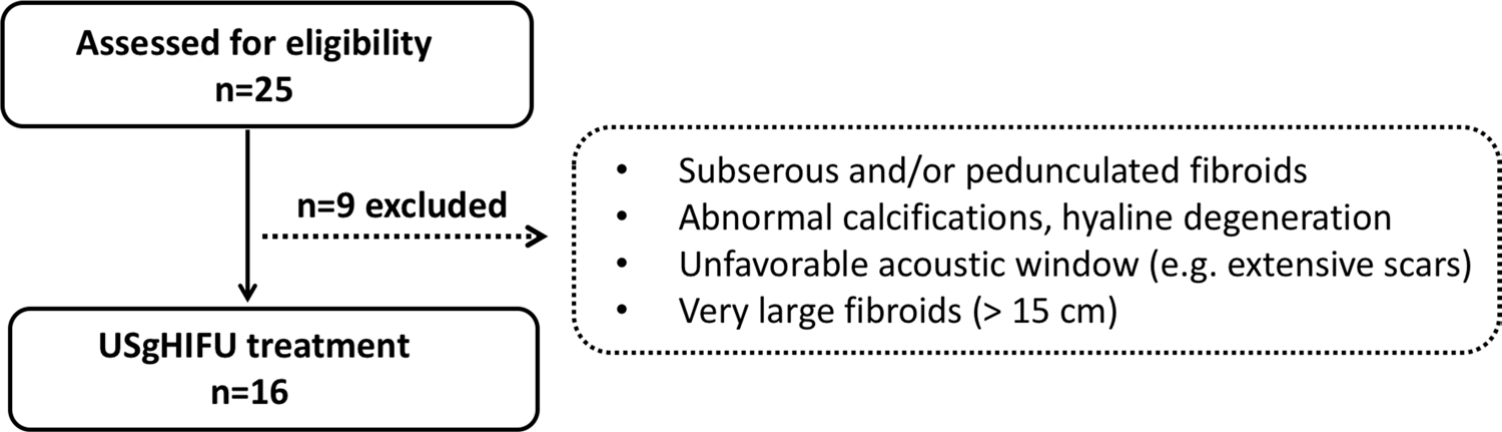
Study consort diagram

The indication for HIFU was made on an interdisciplinary basis and took into account factors such as clinical condition, symptoms and the patient’s individual circumstances. Eligibility for US-guided HIFU treatment was assessed using diagnostic US and magnetic resonance imaging (MRI) of the pelvis (Fig. 1). The criteria for inclusion and exclusion are outlined in Table 1. The study was conducted in accordance to the Declaration of Helsinki. It was approved by the local ethics committee of the University Hospital Bonn (No. 295/19), and it was registered in the German Clinical Trials Register (DRKS00020529). Written informed consent to participation was obtained from all patients.

**Table 1:** Selection criteria for US-guided HIFU with the portable Mirabilis system*

Inclusion criteria	Exclusion criteria
Age ≥ 18 years	Current pregnancy or breastfeeding
Written informed consent	Suspected malignancy
Ability to follow study instructions and participate in required study visits	Abnormal cervical cancer screening result
Disease-related symptoms	Acute infection (e.g., cystitis, pneumonia)
Fibroid visualization on diagnostic US*	Pedunculated or subserosal fibroids
Minimum fibroid diameter of 3 cm	Thick scar tissue on the skin or in the acoustic pathway
Maximum fibroid diameter of 12 cm	Non-eligibility for conscious sedation
Safe acoustic access path to the fibroid	
Distance between skin surface and deepest fibroid regions of max. 9 cm	
Patient`s suitability for MRI*	

^*^HIFU, High-intensity focused ultrasound, * MRI*, magnetic resonance imaging, *US*, ultrasound

### US-guided HIFU treatment

**Fig. 2:**
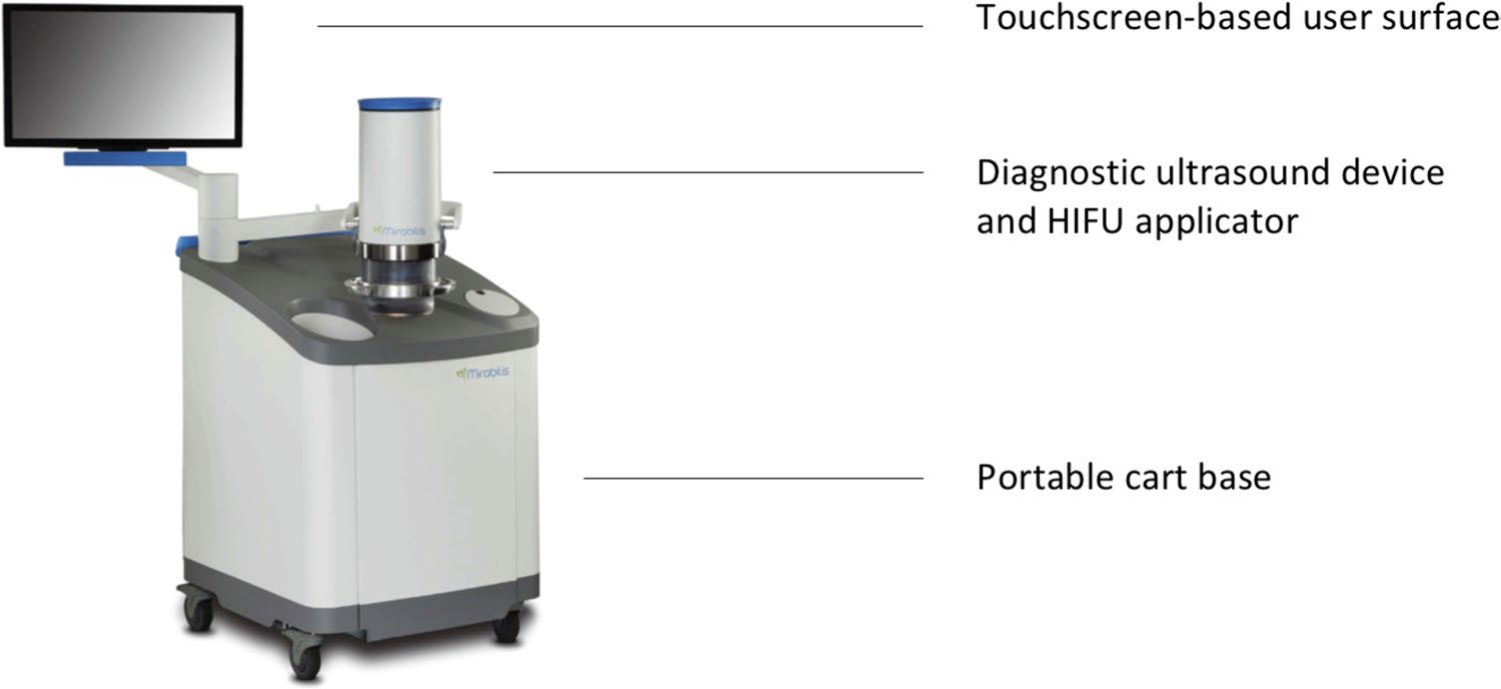
Design of the Mirabilis system for rapid noninvasive HIFU ablation (Mirabilis Medical, Inc., Bothell, Washington, USA). The device consists of a portable cart base with a movable unit that is fixed to a mechanical arm and contains a diagnostic US device (BK Medical Model 2300; Analogic Corporation, Peabody, MA, USA) and a HIFU transducer. A touchscreen-based user interface displays diagnostic US images and allows controlling of the HIFU applicator and adjustment of treatment parameters. HIFU: high-intensity focused ultrasound, US: ultrasound

**Fig. 3:**
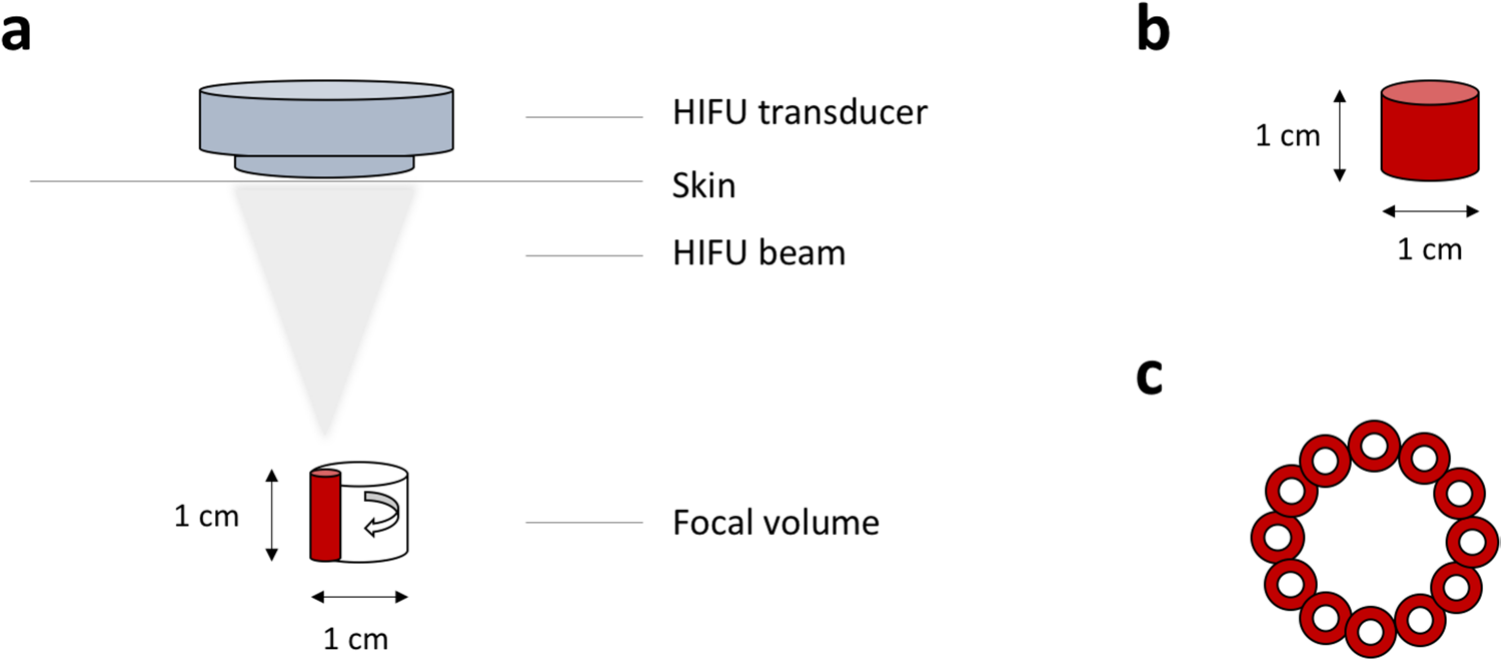
Volumetric shell ablation technique used by the Mirabilis system. **a, b** The extracorporal HIFU transducer generates a HIFU beam creating a focus with a length of 10 mm and a width of a few millimeters. Automatic rotation of the HIFU beam forms a cylinder-shaped unit volume. **c** Top view of multiple unit volumes that are created in the periphery of the target fibroid successively in shape of one or more rings to form a shell (maximum diameter: 5 cm, depth range: 3.5 – 9.5 cm). HIFU: high-intensity focused ultrasound

The ablation of uterine fibroids was performed using the portable Mirabilis system for transabdominal US-guided HIFU (Mirabilis Medical, Bothell, WA, USA). The system comprises a mobile base cart with a cylindrical unit consisting of a diagnostic US probe (BK Medical Model 2300; Analogic Corporation, Peabody, MA) and a therapeutic HIFU transducer (Fig. 2). The therapeutic HIFU transducer is a hemisphere-shaped, multi-element piezoelectric ceramic with a fixed mechanical focus of 12.5 cm from the transducer face with a center frequency of 1.02 MHz. For manufacturing purposes, the therapeutic HIFU transducer consists of 16 elements; all elements are driven simultaneously. The HIFU transducer contains an oculus in the center to accommodate the diagnostic US imaging probe so that the HIFU and imaging beams are axially aligned. The therapeutic HIFU transducer is moved in the X, Y and Z axes by a high-speed motorized assembly which mechanically steers the HIFU beam. Mechanically moving the HIFU beam eliminates focusing degradation typically associated with electronic beam steering. In addition, the device is equipped with a separate screen-based control unit. The Mirabilis HIFU system utilizes the ‘shell ablation’ technique, which involves the thermal coagulation of peripheral penetrating arteries simultaneously at multiple adjacent points, effectively creating a continuous wall (or shell) of devitalized tissue which results in ischemia of the fibroid tissue within the shell (Fig. 3). Users can select from ten preprogrammed treatment sizes (ranging from 1.5 to 3.5 cm in height and 3.0 cm to 5.0 cm in diameter) and also adjust depth of treatment. However, some treatment parameters such as output power, pulse repetition frequency and duty cycle cannot be adjusted by the user, and preset HIFU treatment parameters had to be used. These were developed and validated by extensive testing including computer modeling, bench testing, and preclinical and clinical testing [19].

The Mirabilis system uses a fixed relationship between acoustic power and the treatment depth, with a range of temporal averaged power of 60-190W, depending on the depth of focus. This range is consistent with the literature which showed that an acoustic output of 140W is adequate to safely ablate fibroid tissue [20]. Real-time US imaging is performed by the integrated diagnostic US device throughout the HIFU procedure, including during the actual HIFU treatment. The Mirabilis system uses proprietary imaging filters which reduce or eliminate HIFU energy related artifacts from the diagnostic US images. Real-time US imaging during HIFU treatment allows the user to ensure that the HIFU focus remains on the fibroid to verify that any patient or user movement does not compromise the targeting.

In preparation for the therapy, a 12-h fast was performed starting the day before the ablation procedure. Prior to the HIFU procedure, the skin in the acoustic pathway was prepared by shaving, degreasing and degassing the anterior lower abdominal wall. The HIFU ablation was performed with the patient in a supine position under conscious sedation. During the ablation, the treatment unit was positioned on the patient’s anterior lower abdominal wall. Then, the target myoma was identified using diagnostic US. To prevent heat-related complications to adjacent structures at risk, a safety distance of 1 cm was maintained between the fibroid margin and surrounding organs. The shell ablation was achieved by performing multiple peripheral ring-shaped sonications. The total treatment time varied between 15 min and one hour depending on target fibroid volumes. Following the HIFU treatment, patients were kept hospitalized for the first night.

Adverse events were recorded during the inpatient stay and at each follow-up time point (1 and 6 weeks, 3, 6 and 9 months, and 1 year after HIFU). In the case of a treatment-related adverse event, patients were contacted at two-week intervals until the event has resolved.

### Follow-up imaging and clinical evaluation

The follow-up imaging consisted of contrast-enhanced MRI (1.5-T Ingenia MRI, Philips Healthcare, Best, the Netherlands) and contrast-enhanced US (CEUS) (EPIQ 5G, Philips Healthcare, Best, the Netherlands). The contrast medium used for CEUS was SonoVue® (Bracco Imaging S.p.A., Milan, Italy).

On MRI, the target fibroids were categorized according to the Funaki classification (type 1–3) by evaluating the signal intensity of the fibroids relative to the intensity of the surrounding myometrium and skeletal muscle on T2-weighted images [21]. Treatment success was evaluated in detail on MRI by measuring the non-perfused volume (NPV) of the target fibroids. Fibroid volumes were evaluated using T1- and T2-weighted MRI. Fibroid volumes were evaluated using T1- and T2-weighted MRI. The first CEUS examination was conducted immediately after the HIFU procedure to assess the real-time response of the fibroid. The first MRI was performed within 3 days post-procedure to evaluate early treatment effects. Subsequent imaging sessions were scheduled at 6 weeks, 3, 6 and 9 months, and 1 year after the HIFU treatment to monitor the midterm outcomes.

Evaluation of symptoms and QOL was performed using the Uterine Fibroid Symptom and Health-related Quality of Life Questionnaire (UFS-QOL) [22–24]. The UFS-QOL consists of 37 questions, with 8 items specifically addressing the symptom severity (symptom severity score, SSS) and 29 items focusing on the patients’ HRQOL. Each item is rated on a Likert scale from 1 to 5 points. The scores from the subscales are then converted into transformed scores for symptom severity and HRQOL, ranging from 1 to 100. A higher SSS indicates more severe symptoms, while a higher score on the HRQOL and its subscales signifies a better QOL. The assessment with the UFS-QOL questionnaire was performed at the same time points as the imaging sessions.

### Statistical analysis

Data were analyzed using Stata version 16 (StataCorp. Stata Statistical Software: Release 16. College Station, TX: StataCorp LP). Descriptive statistics such as mean, median, standard deviation (SD), range and 95% confidence intervals (CI) were calculated to summarize data. The statistical evaluation of fibroid volumes and subscales of SSS and HRQOL was performed using the mixed longitudinal (panel) model at baseline and each follow-up as dependent variables. The results are presented as contrasts (differences) and verified for susceptibility of model dependency using the nonparametric Skillings–Mack test for unbalanced panel data. A *p*-value of < 0.05 was considered statistically significant.

## Results

### Patients

From 2020 to 2021, 16 patients with a total of 23 symptomatic uterine fibroids (Table 2) were successfully treated in a single session by US-guided HIFU using the portable Mirabilis system.

**Table 2:** Clinical characteristics of Mirabilis-treated patients with symptomatic uterine fibroids

Parameter	
Patients (no.)	16
Age (years)	44.8 ± 6.7 (32–54)*
Treated uterine fibroids (no.)	23
Treated fibroids per patient (no.)	
1	11 (68.8%)
2	3 (18.8%)
≥ 3	2 (12.5%)
Average fibroid volume (cm^3^)	145 ± 183.1 (3.3–656)*
Location of the target fibroid	
Partially submucosal	3 (13%)
Intramural	18 (78.2%)
Partially subserosal	2 (8.7%)
Funaki type	
Type 1	6 (26%)
Type 2	14 (60.9%)
Type 3	3 (13%)
Symptom severity	
Mild	2 (12.5%)
Moderate	10 (62.5%)
Severe	4 (25%)

^*^Mean ± standard deviation (minimum–maximum range)

Eleven patients (68.8%) had a solitary fibroid, and 5 patients (31.2%) had two or more fibroids and were all treated within a single session. Among the ablated fibroids, the majority (n = 18; 78.2%) were predominantly located intramurally, 3 fibroids were located partially submucosal (13%) and 2 fibroids were located partially subserosal (8.7%). Most fibroids were assigned Funaki type 2 (n = 14; 60.9%), 6 fibroids (26%) were categorized type 1 (26%) and 3 fibroids were assigned type 3 (13%).

### Follow-up imaging and fibroid volume reduction

**Fig. 4:**
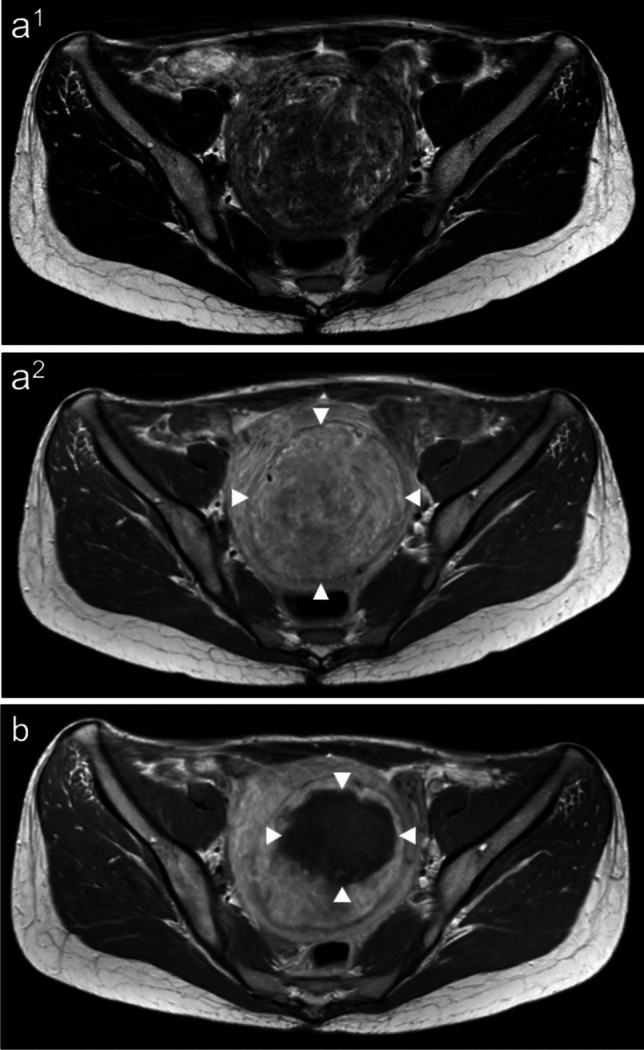
MRI images of a 40-year-old premenopausal patient with multiple fibroids. The patient presented with severe fibroid-associated symptoms (hypermenorrhea, pelvic pain and urinary urgency). There was no fibroid-specific previous therapy. The largest fibroid was located in the posterior wall of the uterus and was the main target of ablation. **a** Large target fibroid of the posterior wall of the uterus before HIFU treatment with a volume of 400.9 ml. The T2-weighted image shows that the target fibroid is partially isointense and partially hyperintense compared to myometrium indicating Funaki type 3. The contrast-enhanced T1-weighted image (c) shows an extensive enhancement of the fibroid (arrows). **b** The contrast-enhanced T1-weighted image shows an extensive enhancement of the fibroid. **c** One day after HIFU treatment. Contrast-enhanced T1-weighted MRI shows a large devascularized ablation volume with a NPV of 41.8%. HIFU: high-intensity focused ultrasound, MRI: magnetic resonance imaging, NPV: non-perfused volume

**Fig. 5:**
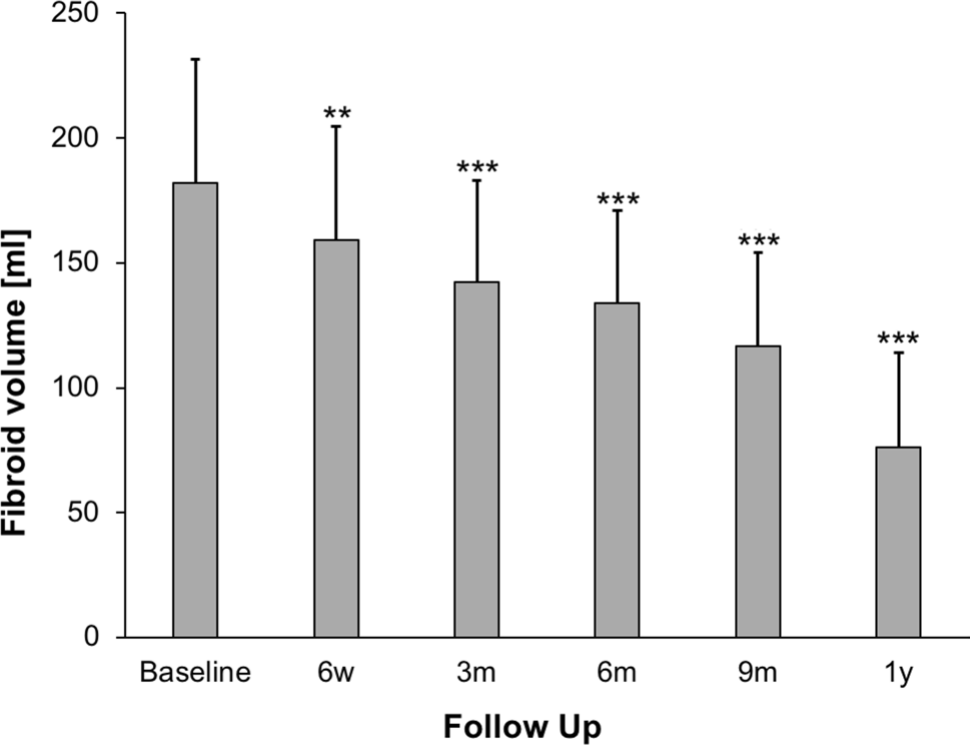
Average reduction of uterine fibroid volume (ml) after US-guided HIFU. Significance levels are indicated by the stars attached (** indicating *p* < 0.01, *** indicating *p* < 0.001). Standard errors were symmetrical (only positive error bars are shown). Significant volume regression was observed at each time point of follow-up between 6 weeks and 1 year compared to baseline. HIFU: high-intensity focused ultrasound, m: month, US: ultrasound, w: week, y: year

The initial follow-up included imaging and clinical examination and was performed within the first few days after HIFU treatment in order to exclude complications such as bleeding, bowel damage or occlusion of periuterine vessels. A devascularized ablation volume was observed in every treated fibroid, even in Funaki type 3 fibroids (Fig. 4). The average non-perfused ablation volume (NPV) of all Funaki types in the first follow-up imaging was 49 ± 9.9% (ranging from 19.7 to 80.0 ml). Notably, the average NPV was higher in fibroids of Funaki type 1 (56.0 ± 23%) and type 2 (56.3 ± 19%) compared to type 3 fibroids (36.8 ± 9%). Significant reduction in fibroid volume was already observed at the 6-week follow-up (11.4 ± 4.1%, p < 0.01). The midterm follow-up imaging at every time point between 3 months and 1 year consistently demonstrated a significant reduction in volume (3 months: 32.2 ± 4.6%, p < 0.001; 1 year: 47.3 ± 6.2%, *p* < 0.001) (Fig. 5).

### Clinical outcome

**Fig. 6:**
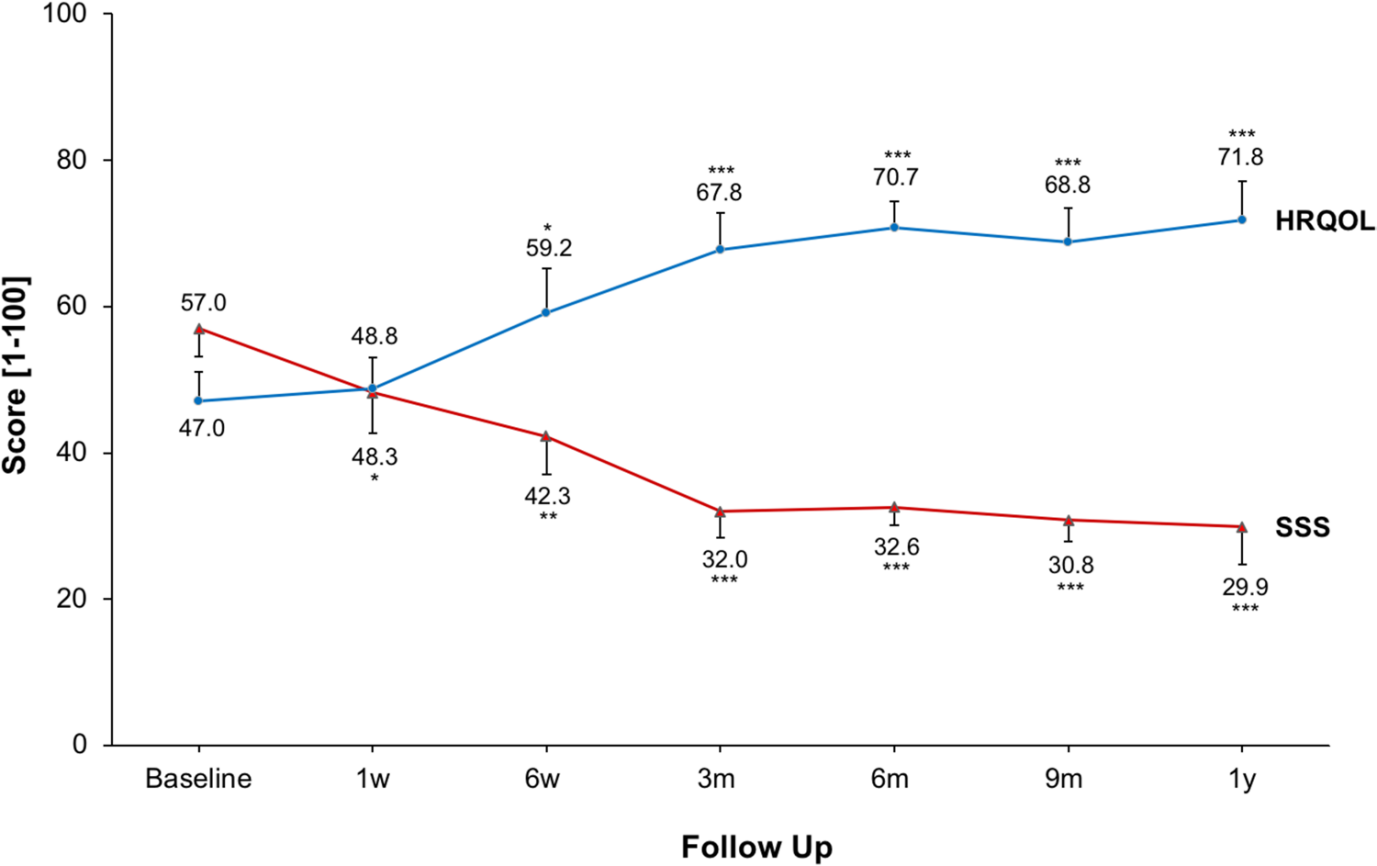
Changes in SSS and HRQOL mean scores of SSS and HRQOL after HIFU are presented. Significance levels are indicated by the stars attached (* indicating *p* < 0.05, ** indicating *p* < 0.01, *** indicating *p* < 0.001). Standard errors were symmetrical (positive error bars shown for HRQOL and negative bars shown for SSS). FU: Follow-up, HRQOL: health-related quality of life, m: month, SSS: symptom severity score, w: week, y: year

After HIFU treatment, all patients reported an improvement of clinical symptoms. During the follow-up period, there was a significant decline in fibroid-associated symptoms and a subsequent improvement in the QOL (Fig. 6). The mean SSS exhibited a significant reduction compared to baseline at both the short-term follow-up of 6 weeks post-HIFU (57.2 ± 3.8 at baseline vs. 42.2 ± 5.2 at 6 weeks, *p* < 0.01) and the midterm follow-up between 3 months and 1 year after therapy (3 months post-HIFU: 31.9 ± 3.6, *p* < 0.001; 1 year post-HIFU: 30.2 ± 4.9, *p* < 0.001). Concurrently, there was a significant increase in HRQOL between 6 weeks and 1 year after HIFU treatment (47.0 ± 3.9 at baseline vs. 71.8 ± 5.3 at 1 year after treatment, *p* < 0.001).

### Adverse events

Following the HIFU procedure, minor self-limiting treatment-associated side effects of short duration were observed. There were no adverse events that occurred during HIFU treatment.

Four patients (25%) reported mild pain in the lower abdomen during treatment, which disappeared immediately after the procedure. Seven patients (43.8%) reported minor vaginal bleeding for a few days following HIFU, which resolved on its own without requiring specific therapy.

In the first post-interventional MRI scan, mild skin edema of the anterior abdominal wall in the acoustic access pathway was observed in 2 patients (12.5%). A slight increase in pelvic fluid compared to baseline imaging was detected in 2 patients (12.5%). However, both the edema of the anterior lower abdominal wall and the pelvic fluid completely regressed by the second follow-up. No long-term treatment-associated side effects were observed.

## Discussion

Uterine fibroids primarily affect women in their reproductive years. US-guided HIFU has emerged as an uterus-preserving treatment option for local fibroid ablation, and it has become an established pillar in the management of symptomatic leiomyomas alongside pharmacological therapy and surgical options [25, 26]. Currently, stationary devices with various US beam delivery systems that can create a single ablation focus are commonly used [25]. Consequently, volume ablation with these devices is achieved by performing multiple focal sonications in rows and adjacent layers, resulting in average treatment time of approximately 2 to 4 h, depending on the number, size and location of the fibroids [25, 26].

The portable mobile US-guided Mirabilis system for HIFU treatment of uterine fibroids offers a novel ablation technique based on simultaneous sonication of multiple peripheral volume units, ensuing in volumetric shell ablation. To the best of our knowledge, this single-center study represents the first reported clinical experience of US-guided HIFU utilizing the Mirabilis system in a European population of women diagnosed with symptomatic uterine fibroids.

Early clinical data from the pilot study of Parsons et al. demonstrated that this new approach allows for effective ablation of fibroid tissue by inducing sufficient non-perfused volumes achieved by strategic simultaneous sonication of small fractions of fibroid tissue, particularly in the myoma periphery. As a result, reduction of fibroid volume over a 6-month period, along with a decrease in symptom severity and an improvement in HRQOL, was observed [19].

In our prospective observational clinical study, the primary objective was to evaluate the safety, feasibility, tolerability and clinical outcomes of the novel mobile HIFU system. With respect to procedure safety, we only observed light adverse events that did not necessitate any specific intervention or treatment. The most side effect was mild pain in the lower abdomen limited to the duration of the procedure (4/16 patients) or minor self-limiting vaginal bleeding (7/16). These findings are in line with previous reported clinical data using different HIFU systems and highlight the general safety profile of HIFU, especially when compared to surgical alternatives [27–30]. A multicenter large cohort study showed an overall incidence rate of major adverse effects of 0.38% after HIFU treatment, while in another study the incidence of significant complications after laparoscopic myomectomy was 3.5% [29].

This high procedure safety resulted in a rapid patient recovery that allowed a short hospital stay as all of our treated patients were discharged from hospital on the day after HIFU treatment. This is in line with previous clinical data showing shorter hospital stays after HIFU compared to conventional uterus-preserving treatment approaches such as laparoscopic myomectomy with a mean difference from 3.3 to 6.9 days [29–33].

When compared to other stationary HIFU systems, the mobile system used in our study offered several advantages: It was easier to handle, allowed for better interaction between physician and patient, and the supine treatment position was more comfortable for the patients as the prone position in stationary systems. Average total treatment time varied between 15 and 60 min and thus was considerably shorter than former treatments at our center with a stationary device (162 ± 47 min), despite larger volumes of fibroids that were treated with the mobile system (mobile system: 145 ± 183.1 ml; stationary system: 109.9 ± 125 ml). However, total treatment times were longer than in the pilot study of Parsons et al. (5.6 ± 2.9 min), due to significantly larger volumes of the target fibroids (145 ± 183.1 ml vs. 22.1 ± 7.7 ml) [19]. Compulsory automatic adjustment of some treatment parameters such as output power, pulse repetition frequency and duty cycle reduced the risk of overtreatment. Another favorable observation concerning the treatment tolerability and peri-interventional clinical management pertains to the use of conscious sedation with a lower dosage of peri-interventional opioid medication compared to prior HIFU treatments with a stationary device, since patient-reported pain during the procedure was less frequent. An important advantage over MRI-guided devices is real-time US imaging reducing risk of insonating bowel or other nontarget tissues and allowing visualization of HIFU-induced echogenic changes (if any) in the target area.

Initial response to treatment was evaluated by measuring the non-perfused volume on the first post-HIFU imaging as objective indicator of ablation outcome. Our results demonstrated a higher rate of successfully treated patients with a detectable NPV on MRI compared to the pilot study (100% vs. 93.2%). Average NPV was 49 ± 19.9%, which was superior to the NPV of 31.2 ± 46.4% reported in the pilot study [19]. When stratifying our results, it seems that NPV was higher in Funaki type 1 (56.0 ± 23%) and 2 fibroids (56.3 ± 19%) than in type 3 fibroids (36.8 ± 9%).

However, recent data from two meta-analyses revealed higher NPV values after HIFU treatment with MRI-guided devices, with mean values of 68.1% and 67.6%, which closely align with the average NPV of 60.1% observed in our clinical experience using a US-guided stationary device [30–32]. Fibroid volume shrank during the follow-up with a continuous significant reduction in fibroid volume between 6 weeks and one year after HIFU treatment compared to baseline. This indicates an early onset of fibroid shrinkage with a sustainable medium-term effect, despite the slightly lower NPV values compared with MRI-guided devices, which may be explained by the systematic peripheral devascularization by ablating small peripheral volume units as part of the shell ablation technique. Notably, all patients in our study underwent a single treatment session, and there were no indications for a re-intervention within the first year, such as significant fibroid growth or recurrence of symptoms during the follow-up period.

The follow-up period in our study extended for 12 months, which was six months longer than the pilot study conducted by Parsons et al. [19]. Our study exhibited a higher fibroid volume reduction rate at 6 months after HIFU (34.2 ± 5.6% vs. 24 ± 42.8%), which may be due to better ablation outcome with higher NPV after treatment. In line with our results, previous clinical reports have shown varying fibroid shrinkage rates at 6 months after MRI- or US-guided HIFU, ranging from 31.7 to 59.1% [27, 33–35]. A recent meta-analysis, including 1323 patients, demonstrated a mean reduction rate of 37.7% at one year after MRI-guided HIFU, which is lower than the reduction rate after one year that we achieved after treatment with the Mirabilis system in our study (47.3 ± 6.2%) [32].

At the same time, HIFU treatment with the Mirabilis system demonstrated a significant reduction in fibroid-associated symptoms, as evidenced by a noteworthy decrease in the symptom severity score and a substantial improvement in HRQOL. In comparison with the pilot study by Parsons et al., our results exhibited a more pronounced effect on both the SSS and HRQOL [19]. Specifically, a significant decrease in SSS was observed at 3 months after HIFU (-40.1 ± 6.9 compared to baseline), whereas the pilot study reported a clinically noticeable but not statistically significant reduction during the same follow-up period (-8.7 ± 19.8 compared to baseline). We observed a significantly greater improvement in HRQOL at 3 months after HIFU (53.9 ± 14.8 compared to baseline) compared to the pilot study (25.6 ± 25.5). Our clinical follow-up period extended also for 12 months, while this was only 3 months in the pilot study. Compared to surgical approaches such as myomectomy and hysterectomy, clinical data have demonstrated a more rapid improvement in HRQOL after HIFU, with similar long-term effects on QOL over one year [27]. In conclusion, the use of shell ablation with the Mirabilis system represents a safe and feasible technique for treating symptomatic uterine fibroids. This approach enables rapid HIFU treatment, even for large myomas, and results in effective fibroid volume ablation. Consequently, it leads to a significant reduction in fibroid volume and alleviation of myoma-associated symptoms, thereby contributing to a notable improvement in the QOL of the patients. This indicates a positive impact on the patients’ overall well-being and quality of life. In addition, the design of the Mirabilis system facilitates easy handling and ensures a high level of patient comfort. However, further research and comparative studies are needed to fully assess the advantages and disadvantages of the mobile HIFU system compared to other treatment options for symptomatic uterine fibroids.

## Data Availability

The data that support the findings of this study are available from the corresponding author upon reasonable request.
